# Prevalence of Intestinal Parasitic Infections and Associated Factors among Presumptive Pulmonary Tuberculosis Patients at Debre Tabor Referral Hospital, South Gondar, Northwest Ethiopia: A Cross-Sectional Study

**DOI:** 10.1155/2024/8993666

**Published:** 2024-05-18

**Authors:** Debaka Belete, Azanaw Amare, Tesfaye Andualem, Desie Kasew, Sirak Biset

**Affiliations:** ^1^University of Gondar, College of Medicine and Health Sciences, School of Biomedical and Laboratory Sciences, Department of Medical Microbiology, Gondar, Ethiopia; ^2^Debre Tabor University, College of Health Sciences and School of Medicine, Department of Medical Laboratory, Debre Tabor, Ethiopia

## Abstract

**Background:**

In developing countries, intestinal parasitic infections (IPIs) and tuberculosis (TB) coinfections have been perceived to be high. The geographic distributions of helminths and TB overlap substantially. Parasitic infections affect the outcome of TB by changing the cell-mediated immune response to a humoral response, while *Mycobacterium* infection favors the immune escape of helminths. There are limited studies on the epidemiology of intestinal parasites among presumptive pulmonary TB (PTB) patients in Ethiopia. Therefore, this study is aimed at determining the prevalence of intestinal parasitic infections and associated factors among patients with presumptive pulmonary tuberculosis at Debre Tabor Referral Hospital.

**Methods and Materials:**

A hospital-based cross-sectional study was conducted from March to June 2021. The sociodemographic data and associated factors were collected using a structured questionnaire, and stool samples were collected by convenient sampling technique and processed for the detection of intestinal parasites using a direct wet mount saline preparation and formal ether concentration technique. The data was coded, cleaned, and analyzed by SPSS version 23. Bivariate and multivariable analyses were conducted to determine an adjusted odds ratio (AOR). *p* value < 0.05 was considered statistically significant.

**Result:**

The overall prevalence of intestinal parasitosis was 25.6% (81/316); of these, 12.9% (41/316) were protozoan infections and 12.7% (40/316) were helminth infections. Multivariable logistic regression analysis showed that being older than 36 years (AOR: 4.35; 95% CI: 1.26, 13.91; *p* = 0.001), rural residence (AOR: 3.46; 95% CI: 1.18, 9.97; *p* < 0.001), unable to read and write (AOR = 2.62; 95%CI = 2.15, 8.43; *p* = 0.004), and use of river water (AOR: 3.47; 95% CI: 1.62, 8.21; *p* < 0.001) were associated with intestinal parasitic infections.

**Conclusion:**

The present study showed that the prevalence of intestinal parasitosis among presumptive pulmonary tuberculosis patients was high in the study area. Age, residence, educational status, and source of water were significant factors in IPIs among presumptive TB patients. Moreover, our findings suggest a proper health education program for good personal hygiene habits, the coloration of water, avoiding open-field defecation, and also preventative measures to avoid the acquisition of IPIs in patients with TB. Presumptive tuberculosis patients should be screened and treated accordingly. Additionally, it needs further research and recommends more assessment for intestinal parasitic infection in PTB patients.

## 1. Introduction

Intestinal parasitic infections (IPIs) are among the major public health problems in developing countries. According to a recent report by the World Health Organization (WHO), about 1.5 billion people (24% of the global population) are infected with IPs [1]. It is estimated that 3.5 billion people are affected globally and 450 million are ill as a result of these infections [2]. Tuberculosis (TB) continues to be one of the most important causes of morbidity and mortality worldwide. It is one of the top 10 causes of mortality with an estimated 10 million new cases each year worldwide [3]. Presumptive pulmonary TB refers to a person with any of the signs and symptoms suggestive of TB including cough > 2 weeks, fever > 2 weeks, unexplained significant weight loss, hemoptysis, or any abnormality in chest X-ray [4]. The TB disease is prevalent in sub-Saharan African countries due to inadequate health infrastructure and other poverty-related factors [5]. Both TB and parasitic diseases in humans are chronic infectious diseases that exhibit an extensive distribution, causing serious harm to humans [6]. Furthermore, TB and IPs primarily affect low social and economic level populations who live in precarious habitation settings [7]. Both IPIs and TB are chronic infectious diseases that cause serious harm to humans [8].

Protozoan infection can be asymptomatic and may result in elevated levels of cytokines such as interferon-*γ* (IFN-*γ*), a critical mediator in the host immune response to *Mycobacterium tuberculosis* [9].

Infections with helminths induce Th2 immune responses, which can include several cytokines such as IL-4, IL-5, and IL-13. As a result, T cell regulation expands over time. This primarily affects the Th1 type of immune response to mycobacterium culture filtrate protein in humans and animal models, returning to normal Th1 responses specific to MTB after the helminth infection has been fully treated [1, 2]. Furthermore, a study done on animal models suggests that some pathogens, such as parasitic coinfection, are linked to more severe bovine tuberculosis (BTB) wounds in wild boar because the prevalence of BTB in wild boar animals has been linked to the availability of animals with more severe BTB wounds. This study revealed a favorable correlation between BTB severities and coinfection with *Metastrongylus* species [10].

The epidemiology of IPIs shows that parasites are found in every age group and both sexes. However, the incidence is high in some areas and some age groups. Parasites interfere with the nutritional and immune profiles of an individual, promoting secondary infections such as *Mycobacterium tuberculosis* (MTB). In sub-Saharan African countries, where TB is also prevalent, the prevalence of IPIs is remarkably high [11, 12].

The majority of intestinal protozoa that inhabit the gastrointestinal tract of humans are nonpathogenic commensals or only result in mild diseases; however, some of them, namely, *Entamoeba histolytica* and *Blastocystis hominis* can cause severe diseases under favorable conditions. Both TB and intestinal parasites, as indicated above, are in great distribution which means both of them look like community or public problem [13].

The increased incidence of intestinal parasites among PTB patients suggested a rise in morbidity, underscoring the significance of ongoing stool testing and prompt treatment for PTB-suspected patients [14, 15].

Furthermore, throughout their life cycle, soil-transmitted helminths (STHs) such as *Ascaris lumbricoides*, *Strongyloides Stercoralis*, and hookworm can migrate from the heart to the lung, causing symptoms that could be mistaken for PTB [16, 17]. Therefore, the appropriate management of PTB-suspected patients must assess IPIs, primarily STH. Also, it is helpful to evaluate the burden of coinfection between PTB and IPIs, as in coendemic areas, coinfection can make it more difficult to prevent and treat PTB and intestinal parasite infections [18, 19].

Tuberculosis and parasitosis are widely distributed diseases in Ethiopia, where they are the leading cause of mortality and morbidity, with 32% of hospitalized TB patients having intestinal parasites and 29% of TB patients from the community having intestinal helminths in Ethiopia [20]. Although there are a few reports on the prevalence of IPIs among presumptive PTB patients in Ethiopia. However, evidence is not enough concerning the extent of PTB and IPI in most of the provinces. Thus, the goal of this study was to determine the prevalence of IPI and associated factors among presumptive PTB patients to provide valuable public health information.

## 2. Methods and Materials

### 2.1. Study Design, Setting, and Period

A hospital-based cross-sectional study was conducted to determine the prevalence and associated factors of intestinal parasitosis among presumptive PTB patients attending Debre Tabor Referral Hospital (DTRH) in South Gondar, Northwest Ethiopia, from March to June 2021. The hospital is a referral-level hospital that serves over three million inhabitants and residents of Debre Tabor City. The city is located in the Gondar Administrative Zone of the Amhara National Regional State, 666 km north of the capital city, Addis Ababa. It has one government hospital, three public health centers, four public health posts, and three private clinics [21].

### 2.2. Source Populations

All presumptive PTB patients who were attending DTRH in South Gondar, Northwest Ethiopia, were used as the source population.

### 2.3. Study Population

All presumptive PTB patients who were attending DTRH in South Gondar, Northwest Ethiopia, from March to June 2021 were used as the study population.

### 2.4. Inclusion Criteria

All patients who had a persistent cough lasting for two weeks or more and were thought to have PTB were included in the study population.

### 2.5. Exclusion Criteria

Participants who started anti-TB treatment and those who had taken helminthic treatments in the two weeks before specimen collection were excluded from the study.

### 2.6. Sample Size Determination and Sampling Technique

The sample size was calculated using the single population proportion formula (*Zα*/2)2(*p*) (1 − *p*)/*d*2, using a prevalence (*p*) of 28.9% from a previous similar study conducted at the University of Gondar Comprehensive Specialized Hospital [22], and we employed a 95% confidence interval and a 5% margin of error accordingly. The total sample size (*n*) calculated was 316. Study participants were selected based on the information we had on the number of presumptive PTB patients attending DTRH in the study period. The monthly average number of clients attending the TB clinic in the previous four months was 640, and the number of patients included in the study was distributed uniformly each week. Accordingly, the estimated number of presumptive PTB patients attending DTRH TB clinic during the study period per week and per day was 40 and 8, respectively. Depending on this analysis, the approximate numbers of patients who were expected to attend a TB clinic during the next three months (March to May 2021) of the study period were determined. Finally, a systematic random sampling technique (*k* = 640/316 = 2) was used to select study participants.

### 2.7. Data Collection and Processing

Using a standardized questionnaire and a face-to-face interview, a trained nurse gathered sociodemographic information and related factors. Before being used in the actual data gathering procedure, the questionnaire was pretested and amended. During the patient visit, data on the patient's sex, age, occupation, education, marital status, monthly income, and other relevant parameters were collected.

### 2.8. Stool Sample Collection and Processing

Patients were provided with a stool cup that was clean, dry, and leak-proof labeled with a unique identification number, and they were asked to bring about 5 g of stool sample. In order to detect intestinal parasites, standard methods consisted of a direct fresh stool smear using normal saline (0.85% NaCl solution) and Lugol's iodine staining for the detection of protozoal trophozoite and cyst, respectively. The preparation was mounted on the microscope and examined using 10x and 40x objectives with the condenser iris closed to give good contrast for the detection and identification of trophozoites and cysts of parasites [23].

For the formalin-ether concentration technique, centrifuge tubes were labeled, and 4 mL of 10% formalin water was dispensed in each tube. Each tube received 1 g of stool specimen emulsified with an applicator stick. Three milliliters of 10% formalin water was added to each tube, mixed well, and shaken. The emulsified feces were sieved into another set of centrifuge tubes using a fine mesh gauze. Four milliliters of diethyl ether were added to each tube. The tubes were covered with a stopper and mixed for 1 minute; the stopper was removed using tissue paper. The tube containing the stool suspension was centrifuged at 3000 resolution for 1 minute. After loosening the layers of fecal debris from the tubes' sides with an applicator stick, the tubes were inverted and the ether, fecal debris, and formalin water were discarded. The sediments remained, and the tubes were returned to their upright position to allow the liquid from the side to drain to the bottom. The bottom of each tube was tapped to resuspend and mix the sediments. The sediments of each tube were transferred to a clean glass slide covered with a cover slip, mounted on a microscope, and examined using the 10x and 40x objectives for the detection and identification of protozoan cysts and helminths's ova/larva of parasites [23].

### 2.9. Data Quality Assurance

A structured questionnaire developed by adapting different peer-reviewed literature was used to assess sociodemographic variables and potential risk factors for IPI. The questionnaire was prepared in English version, translated into the local language (the Amharic version), and then transcribed back to English to maintain its consistency. To ensure data quality, 5% of the questionnaire was pretested before the actual data collection process.

Quality control methods (preanalytical, analytical, and postanalytical) were used and followed throughout the laboratory testing process to ensure the correctness of the study's findings. Processes, tools, and materials were all effectively managed. Using negative and positive control slides, the laboratory staff and the microscope's performance were both evaluated for accuracy. Two independent reviews of each slide confirmed the result. All fresh stool samples were evaluated within 30 minutes of collection, and in the event of a delay, the protozoan morphology and growth of various helminth eggs and larvae were stopped using the proper preservatives. Instruments underwent preventive maintenance, and the right processing of quality control materials was done.

### 2.10. Data Analysis and Interpretation

The collected data was checked for completeness, and then, the data was entered into SPSS version 23. To summarize the data, statistical analysis for descriptive statistics of the variables was computed. For categorical variables, frequencies and percentages were computed, while for continuous variables, the mean and standard deviation were calculated. The odds ratios (OR) with 95% confidence intervals (95% CI) were calculated. All variables with a *p* value < 0.2 (to control the effect of confounding) in the bivariate analysis were included in the multivariate logistic regression [24]. In all cases, a *p* value ≤ 0.05 was taken as a statistically significant association. Finally, the findings were represented with texts and tables.

### 2.11. Ethical Consideration

The ethical approval was obtained from the Debre Tabor University, College of Health Sciences and Department of Medical Laboratory Science, Research and Ethical Review Committee (permission letter's reference number: CHS/221/2013 in the Ethiopian calendar, date 7/2/2013 E.C). All eligible study participants were informed about the purpose of the study; however, the participants were given the full right to withdraw at any time from participating in the research process. Written informed consent was obtained from each study participant. All stool examination-positive laboratory results were only available to the clinician who attended the patient, and based on the results, the patient was treated accordingly.

## 3. Results

### 3.1. Sociodemographic Characteristics of Study Participants

In this study, a total of 316 presumptive pulmonary TB patients were involved in the study, with a response rate of 100%. In more than half of the study participants, 164 (51.9%) were females, 152 (48.1%) were males, and the majority (150, 47.5%) of the presumptive PTB patients were >36 years. In the majority of study participants, 174 (55%) were rural dwellers and 166 (52.5%) completed primary school. Out of 242 study participants, 163 (51.6%) have monthly income > 3000 birr whereas the rest 153 (48.4%) have monthly income < 3000 birr. For the case of marital status, 171 (47.8%) were married and 52 (16.5%) were single (Tables 1 and 2).

### 3.2. Prevalence of Intestinal Parasitosis

In this study, the overall prevalence of intestinal parasites was 25.6% (81/316). While analyzing the different types of IPs among presumptive PTB patients, out of a total of 316 patients, 41 (12.9%) had intestinal protozoan infections and 40 (12.7%) had intestinal helminth infections. Intestinal protozoan infections *Giardia lamblia* and *Entamoeba histolytica/dispar* were the most frequently detected parasites, with prevalence rates of 22/81 (6.9%) and 19/81 (6%), respectively, while the most frequently detected intestinal helminth infection was *Strongyloides stercoralis* 10/81 (3.2%) followed by hookworm 8/81 (2.5%), *Taenia* species 7/81 (2.2%), *Ascaris lumbricoides* 6/81 (1.9%), *Trichuris trichiura* 5/81 (1.6%), and *H. nana* 4/81 (1.3%) (Table 3).

### 3.3. Factors Associated with Intestinal Parasites among Presumptive PTB Patients

In this study, bivariable and multivariable binary logistic regression were used to determine the associated factors of IPIs. In bivariable binary logistic regression, age groups > 36 years, residence, and educational status were significantly associated with the presence of IPIs. However, individuals' sex, occupation, and marital status were not statistically significant. In bivariate binary logistic regression analysis, of the potential associated factors, handwashing habit after defecation and water source were found to be statistically associated with IPI prevalence (*p* value < 0.05).

After adjusting for factors with a *p* value of 0.2 in the bivariate binary logistic analysis, a multivariable logistic analysis was performed.

A multivariable logistic regression analysis revealed that those older than 36 were 4.35 times (AOR: 4.35; 95% CI: 1.26, 13.91; *p* = 0.001) more likely to develop IP than those younger than 36. Residence was found to have a significant relationship with intestinal parasitic infection, with rural residents being 3.46 times (AOR: 3.46; 95% CI: 1.18, 9.97; *p* < 0.001) more likely to be infected than urban participants. Additionally, there was statistical significant association between IPI and participants' educational status. People unable to read and write had a 2.62 times (AOR = 2.62; 95%CI = 2.15, 8.43; *p* = 0.004) higher risk of developing IP compared with those who had completed primary school and above, and also, use of river water was significantly associated with IPI, with 3.47 times higher infection rate in river water users (AOR: 3.47; 95% CI: 1.62, 8.21; *p* < 0.001) compared with those who used pipe water source (Table 4).

## 4. Discussion

Intestinal parasites [25] and TB are significant health concerns in the majority of developing countries because they cause severe morbidity and disproportionately affect the poorest communities. In this study, the overall prevalence of IPI among presumptive PTB patients was 25.6%. This finding was comparable to studies conducted in Arba Minch, Ethiopia (26.3%) [26], and systematic and meta-analysis reviews in Iran (26%) [27]. However, the result of this study was higher than studies conducted in Gondar, Ethiopia (19.6%) [15]; Adama, East Shewa, Ethiopia (21.4%) [7]; China (14.9%) [28]; and Brazil (19.6%) [29]. On the other hand, these findings were lower than the study conducted in Gondar (28.9% and 40.5%) [14, 22]; Woldia, Ethiopia (49%) [30]; Northwest Ethiopia (40% and 70.9%) [31, 32]; and India (27.1%) [25]. This variation might be due to differences in geographic location, socioeconomic status, sample size, testing methods, and study population.

In this study, intestinal protozoan infections (41, 12.9%) were the most frequently detected among presumptive TB patients. This is lower than the study conducted in Kuyu General Hospital, North Shewa, and Addis Ababa, Ethiopia [20, 33]. This difference might be due to differences in low-level personal hygiene practices such as not washing hands after defecation and a lack of awareness about the prevention and control of intestinal parasitic infection, which are independently associated with parasitic infection.

In this study, the most frequently prevalent intestinal protozoan infections were *Giardia lamblia* (6.9%) and *Entamoeba histolytica/dispar* (6%). This result is in agreement with studies in Gondar, Ethiopia [15]; Sanja, Ethiopia [24]; and Arba Minch, Ethiopia [26]. However, the finding of this study is higher than the study conducted in China [28]. This may be caused by variations in the availability of water, variations in the economy, dietary habits, hygienic conditions in the environment, and knowledge about the modes of transmission, preventative measures, and management strategies for these parasitic diseases.

In the current study, helminth infection accounts for 12.7% of presumptive PTB patients. Among these, the most prevalent intestinal helminth infection was *Strongyloides stercoralis* (3.2%) followed by hookworm (2.5%), *Taenia* species (2.2%), and *Ascaris lumbricoides* (1.9%). However, our studies are discordant with other reports where hookworms were the most frequent helminths in China, Northwest Ethiopia, and Kenya with rates of 13.1%, 4.3%, and 11.1% [22, 34, 35], respectively, among intestinal helminths.

The current study has also shown factors associated with IPIs among presumptive PTB patients. The prevalence of IPIs showed a significant association with different age groups. The age group of >36 years were 4.35 times more likely to be infected with IPIs than those younger than 36 years old. This finding is the concordant study conducted in Gondar, Ethiopia, where the prevalence of IPIs showed a significant association with different age groups. The age group of 19–45 years was the most affected [15]. However, this finding contradicts the findings of an Indian study, which found that rates of intestinal parasitic infection (IPI) were higher in the 11-20-year age group [25]. The reasons could include playing in soil and water, interacting with each other frequently, crammed classrooms, and their early involvement in farming, which promotes the parasites' spread.

The current investigation showed that the participant's level of education was statistically associated with IPI. Individuals who are unable to read and write were 2.62 times more likely to be infected with IPIs than those who had completed primary school and above (AOR = 2.62; 95%CI = 2.15, 8.43; *p* = 0.004). This is in line with other studies conducted in Sanja, Ethiopia [24]; in Bahir Dar, Ethiopia [36]; and in North-Western Nigeria [37]. This result could be explained by the fact that illiterate people are less knowledgeable about IPI preventive and control strategies. Higher education is typically linked to better hygiene and sanitation practices, which may help to lower the prevalence of IPI.

### 4.1. Limitations of the Study

This study has its own limitations. For example, the prevalence of intestinal parasites was determined by the examination of a single stool specimen from each study participant. The Kato-Katz technique was not used to assess the parasite load. The modified Ziehl-Neelsen acid fast staining method was not performed to detect the *Cryptosporidium* and isospora belli oocysts. In addition, the cross-sectional nature of the study did not show cause-and-effect relationships between intestinal parasites and PTB infection. We did not use molecular methods, such as PCR, to distinguish between *E. dispar* and the real pathogenic *E. histolytica*. So it needs cohort studies to be conducted.

## 5. Conclusions and Recommendations

In this study, the overall prevalence of intestinal parasitosis among presumptive PTB patients was high as compared to the previous reports. The most prevalent protozoan infection was *Giardia lamblia* followed by *Entamoeba histolytica/dispar*, while the most prevalent helminth infection was *Strongyloides stercoralis* followed by hookworm, *Taenia* species, *Ascaris lumbricoides*, and *Trichuris trichiura.* Among risk factors of IPIs analyzed, being older than 36 years old, rural residents, unable to read and write, and use of river water showed significant association with intestinal parasite infections. Moreover, our findings suggest a proper health education program for good personal hygiene habits, the coloration of water, avoiding open-field defecation, and also preventative measures to avoid the acquisition of IPIs in patients with TB. Presumptive tuberculosis patients should be screened and treated accordingly. Additionally, it needs further research and recommends more assessment for intestinal parasitic infection in PTB patients.

## Figures and Tables

**Table 1:** Sociodemographic and behavioral characteristics of presumptive PTB patients attending Debre Tabor Referral Hospital, South Gondar, Ethiopia, 2021 (*n* = 316).

Variables	Frequency (*N*)	Percent (%)
Sex		
Male	164	51.9
Female	152	48.1
Age		
18-25	74	23.4
25-35	92	29.1
>36	150	47.5
Residence		
Urban	142	44.9
Rural	174	55.1
Marital status		
Married	171	54.1
Single	52	16.5
Divorced	81	25.6
Widowed	12	3.8
Educational status		
Un able read and write	76	24.1
Primary school	166	52.5
Secondary school	47	14.9
Collage and above	27	8.5
Occupational status		
Governmental employee	44	13.9
Housewife	73	23.1
Private work	32	10.1
Farmer	44	28.9
Other	33	21.7
Monthly income		
>3000 birr	163	51.6
≤3000 birr	153	48.4

**Table 2:** Behavioral and clinical characteristics of the presumptive PTB patients attending at Debre Tabor Referral Hospital, South Gondar, Ethiopia, 2021 (*n* = 316).

Variables	Frequency (*N*)	Percent (%)
Do you always wear shoes?		
Yes	146	46.2
No	170	53.8
Where is the water source		
Spring	123	39
Pipe	142	45
River	51	16
Do you have a latrine		
Yes	188	59.5
No	128	40.5
The habit of eating raw meat		
Yes	165	52.2
No	151	47.8
The habit of eating unwashed vegetables		
Yes	47	14.5
No	269	85.5
Handwash habit before and after meal		
Yes	305	96.5
No	11	3.5
Handwash habit after defecation		
Yes	29	9.2
No	287	90.8
History of diarrhea in the last month		
Yes	136	43
No	180	57
Do you know HIV status		
Yes	123	38.9
No	193	61.1
History of previous PTB treatment		
Yes	4	1.3
No	312	98.7

**Table 3:** Distribution of intestinal parasite species among presumptive PTB patients at Debre Tabor Referral Hospital, South Gondar, Ethiopia, 2021 (*n* = 316).

Intestinal parasite	Frequency (*N*)	Percent (%)
*Ascaris lumbricoides*	6	1.9
Hookworm	8	2.5
*Trichuris trichiura*	5	1.6
*Taenia* species	7	2.2
*H. nana*	4	1.3
*Strongyloides stercoralis*	10	3.2
*Giardia lamblia*	22	6.9
*Entamoeba histolytica/dispar*	19	6
Total	81	25.6

**Table 4:** Analysis of associated factors for intestinal parasitosis among presumptive PTB patients visiting Debre Tabor Referral Hospital, South Gondar, Ethiopia, 2021 (*n* = 316).

Variables	Intestinal parasitic infection (*N*)			COR (95% CI)	*p* value	AOR (95% CI)	*p* value
	Positive	Negative	Total				
Age							
18-25	7	67	74	1			
26-35	27	65	92	0.29 (0.03, 4.12)	0.56	0.62 (0.31, 5.47)	0.71
>36	47	103	150	3.15 (1.75, 9.82)	0.004^∗^	4.35 (1.2, 13.91)	0.001^∗^
Residency							
Urban	37	105	142	1			
Rural	44	130	174	2.81 (1.64, 7.12)	0.004^∗^	3.46 (1.18, 9.97)	0.001^∗^
Educational status							
Unable to read and write	25	51	76	1.80 (0.82, 3.62)	0.17	2.62 (3.15, 8.43)	
Primary school	22	144	166	1.57 (0.31, 4.51)	0.87	0.78 (0.51, 5.12)	0.004^∗^
Secondary school	18	29	47	1.21 (0.72, 2.37)	0.62	1.23 (0.39, 2.91)	
Collage and above	16	11	27	1			
Handwashing							
Yes	23	6	29	1			
Habit after defection							
No	58	229	287	1.38 (0.72, 2.57)	0.31		
Water source							
Spring	30	93	123	1.02 (0.41, 1.54)	0.43	1.37 (0.94, 2.75)	0.86
River	47	4	51	2.02 (1.15, 3.82)	0.001^∗^	3.47 (1.62, 8.21)	0.001^∗^
Pipe	4	138	142	1			

CI: confidence interval; COR: crude odds ratio; AOR: adjusted odds ratio. ^∗^Statistical significance.

## Data Availability

All data is available in the manuscript.

## Abbreviations

AFB: Acid fast bacilli
AOR: Adjusted odds ratio
CI: Confidence interval
COR: Crude odds ratio
DTRH: Debre Tabor Referral Hospital
HIV: Human immune deficiency virus
IP: Intestinal parasites
IPI: Intestinal parasitic infection
MTB: *Mycobacterium tuberculosis*
PTB: Pulmonary tuberculosis
SPSS: Statistical Package for Social Sciences
WHO: World Health Organization.
